# Coronary artery bypass grafting for coronary artery anomalies in infants and young children

**DOI:** 10.1093/icvts/ivac119

**Published:** 2022-05-04

**Authors:** Yu Hohri, Masaaki Yamagishi, Yoshinobu Maeda, Satoshi Asada, Hisayuki Hongu, Satoshi Numata, Hitoshi Yaku

**Affiliations:** 1 Department of Cardiovascular Surgery, Kyoto Prefectural University of Medicine, Kyoto, Japan; 2 Department of Paediatric Cardiovascular Surgery, Children’s Medical Centre, Kyoto Prefectural University of Medicine, Kyoto, Japan

**Keywords:** Coronary artery bypass grafting, Congenital coronary artery anomalies, Paediatric cardiac surgery, Internal thoracic artery, Coronary revascularization

## Abstract

**OBJECTIVES:**

Coronary artery bypass grafting (CABG) has been reported for coronary artery diseases in patients with Kawasaki disease and coronary artery complications after arterial switch operations for transposition of the great arteries. However, only a few studies have explored this modality for congenital coronary artery anomalies. As congenital coronary artery anomalies, particularly left coronary artery atresia and stenosis, are one of the reasons for sudden death, coronary revascularization is often required in infants and young children. Therefore, we aimed to investigate the outcome of CABG for such anomalies in infants and young children.

**METHODS:**

From 2014 to 2018, 3 infants and 2 children (median age: 10 months; range: 6–40 months) with coronary artery anomalies underwent CABG at our hospital. The indications for the procedure included left main coronary artery atresia and stenosis in 2 and 3 patients, respectively. Graft patency was evaluated postoperatively by contrast-enhanced computed tomography or coronary angiography, and postoperative outcomes (including death and cardiac events) were assessed during the follow-up period.

**RESULTS:**

No 30-day or in-hospital mortalities were noted. Postoperative examinations revealed patent grafts in all patients. They were discharged without any cardiac complications. Regarding the outcomes at the follow-up period, the graft patency rate was 80.0% (4/5 grafts), with no deaths or cardiac events.

**CONCLUSIONS:**

CABG is a useful strategy for coronary revascularization in infants and young children with coronary artery anomalies. Although the mid-term outcomes and patency are satisfactory, careful follow-up is necessary because the long-term outcomes remain unknown.

**Figure:**
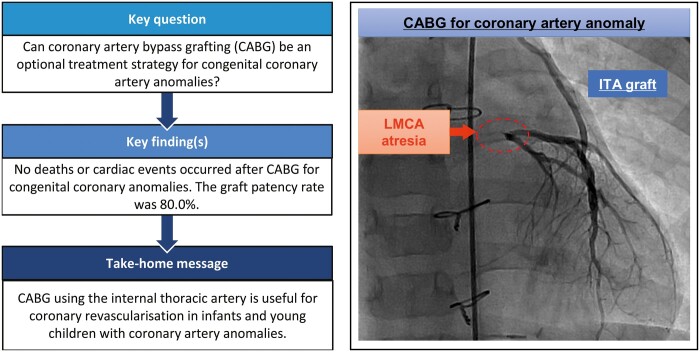

## INTRODUCTION

Previous studies have shown that coronary artery bypass grafting (CABG) can be considered a valuable strategy for coronary revascularization in patients with Kawasaki disease [1, 2] and in those with coronary complications after an arterial switch operation for transposition of the great arteries [3, 4]. However, only a few studies have explored CABG as a treatment option for congenital coronary artery anomalies in young children. Consequently, in cases involving CABG for such anomalies, surgical strategies are based on limited data presented in case reports and small case series [5–7]. Moreover, the follow-up data regarding the outcome of coronary revascularization in infants and young children with coronary artery anomalies are inadequate. Therefore, it is necessary to investigate the outcomes of coronary revascularization in such patients to identify the optimal treatment for these anomalies. In this study, we aimed to investigate the outcomes of CABG for congenital coronary artery anomalies in young children.

## PATIENTS AND METHODS

### Ethical statement

This retrospective study was approved by the institutional ethics committee of the Kyoto Prefectural University of Medicine and conducted in accordance with the principles stated in the Declaration of Helsinki. Written informed consent was obtained from the guardians of all the participating patients.

### Patients

From 2014 to 2018, 3 infants and 2 children with coronary artery anomalies underwent intracardiac repair and CABG at the Kyoto Prefectural University of Medicine. The patients’ age at operation ranged from 6 to 40 months (median months: 10 months). The patient characteristics and diagnoses are presented in Table 1. Two female and 3 male patients were included. One of the patients had previously been diagnosed with Williams syndrome (Table 1; Case 1), and another patient had been diagnosed with duplication of the chromosomal region 9q33.3–q34.11 (Table 1; Case 4). During foetal diagnosis, 1 patient (Table 1; Case 1) was suspected of having valvular heart disease, which was confirmed by transthoracic echocardiography after birth. In the other patients, valvular heart disease was detected by transthoracic echocardiography, which was performed for heart murmur detected at regular health check-ups for infants (Table 1; Cases 2–5). In all cases, coronary artery anomaly was detected by cardiac catheterization or computed tomography, which were performed as part of a complete examination. The indication for CABG was left main coronary artery (LMCA) stenosis in 3 patients and LMCA atresia in 2 patients. In Case 4, the patient presented with shortness of breath (Table 1). However, in almost all cases of infants and young children, it was difficult to notice any symptoms due to ischaemic cardiomyopathy. Therefore, we determined the indication for CABG via physiological ischaemic phenomena during myocardial perfusion scintigraphy. In 2 cases, the indications for coronary revascularization were absolute because of myocardial hypoperfusion (Table 1; Cases 4 and 5). However, the other 3 cases exhibited so-called balanced myocardial ischaemia, in which stress myocardial perfusion scintigraphy showed no resting or stress-induced perfusion defects due to LMCA disease, although ST-segment depression was noted via electrocardiography during stress (Table 1; Cases 1–3), which served as an indication for CABG.

**Table 1:** Preoperative and operative data of the patients

Patient	Age (months)	BSA (m^2^)	BW (kg)	Diagnosis	Coronary lesion	Surgical procedure	CPB time (min)	ACC time (min)
1	6	0.31	6.5	SAS, Williams syndrome	LMCA stenosis	SAS release CABG (LITA-LAD)	288	137
2	9	0.4	8.6	SAS, bicuspid AV	LMCA stenosis	SAS release, AVP, CABG (LITA-LAD)	216	152
3	10	0.39	8.2	AS	LMCA stenosis	Ross procedure, CABG (LITA-LAD)	280	147
4	40	0.62	15.6	9q33.3–q34.11 duplication MR	LMCA atresia	MVP, CABG (LITA-LAD)	110	64
5	19	0.43	9.5	MR	LMCA atresia	MVP, CABG (LITA-LAD)	177	128

ACC: aortic cross-clamp; AS: aortic valve stenosis; AV: aortic valve; AVP: aortic valve repair; BSA: body surface area; BW: body weight; CABG: coronary artery bypass grafting; CPB: cardiopulmonary bypass; LAD: left anterior descending artery; LITA: left internal thoracic artery; LMCA: left main coronary artery; MR: mitral valve regurgitation; MVP: mitral valve repair; SAS: supravalvular aortic stenosis.

### Surgical procedure

In each case, a median sternotomy was performed, and the left internal thoracic artery (LITA) was harvested using an electrocautery instrument rather than an ultrasonic scalpel. The LITA was used as a pedicle and *insitu* graft in all patients. Surgery was performed under cardiopulmonary bypass with arterial cannulation via the ascending aorta, as well as bicaval cannulation, and cardioplegic arrest was established. During CABG, LITA-coronary artery anastomosis was performed using running continuous 8-0 polypropylene sutures (PROLENE, Ethicon Inc., Raritan, NJ, USA). Based on what was appropriate for each case, either a side-to-side or an end-to-side anastomosis was performed. All patients also underwent an LITA-left anterior descending artery bypass. Moreover, concomitant procedures were performed in all patients (Table 1).

### Follow-up

The follow-up clinical data were obtained for all patients by reviewing the medical records from initial admission until the last follow-up examination, and the median follow-up duration was 4.2 (range: 2.3–6.8) years. Currently, there are no recommendations for the performance of postoperative CABG in infants and young children because of limited clinical experience. Therefore, our postoperative follow-up treatment was based on the limited previous studies [2, 3]. Postoperatively, all patients received aspirin and were evaluated routinely for clinical signs of myocardial ischaemia by echocardiography and electrocardiography after discharge from the hospital. Contrast-enhanced computed tomography or coronary angiography was performed in all patients as follows: preoperatively, within 1 month postoperatively, within 1–2 years postoperatively and in the event of cardiac episodes, when the patient’s guardian agreed to the examinations and the patient did not exhibit any allergies to the contrast medium. Graft patency was determined based on these examinations. Cardiac events included death from any cause, acute myocardial infarction, syncopal episodes, angina pectoris, ventricular tachyarrhythmias and events that necessitated further intervention.

### Statistical analyses

Quantitative variables are expressed as medians with ranges and categorical variables as frequencies and percentages. The left ventricular ejection fraction (LVEF) was compared using a paired sample *t*-test. A *P*-value of <0.05 (2-sided) was considered statistically significant. All data were retrospectively analysed and all statistical analyses were performed using JMP^®^ software (version 13.2.0.; SAS Institute, Cary, NC, USA).

## RESULTS

**Figure 1:**
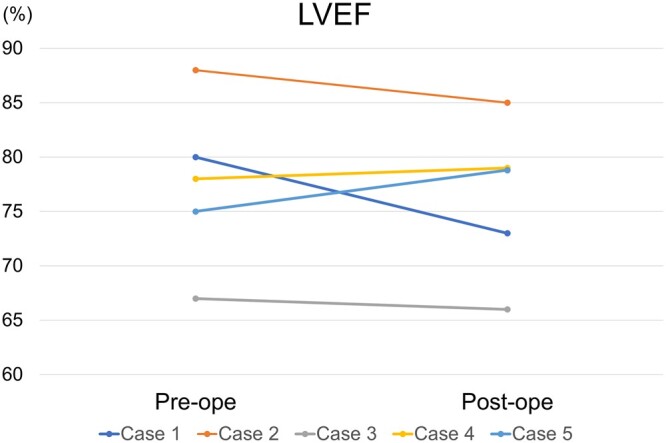
Comparison of preoperative and postoperative LVEF. LVEF was not significantly different between the preoperative and postoperative stages. LVEF: left ventricular ejection fraction.

No 30-day and in-hospital mortalities were observed. Moreover, all grafts were found to be patent during the postoperative examinations before discharge in all patients (postoperative graft patency rate: 100%). All patients were discharged without any cardiac complications, such as perioperative myocardial infarction. There was no improvement in the postoperative LVEF values compared to the preoperative values (preoperation: median: 77.6%, range: 67.0–88.0%; postoperation: median: 76.4%, range: 66.0–85.0%, *P* = 0.53; Fig. 1).

Regarding the follow-up outcomes, no deaths or cardiac events occurred during the follow-up period. Although coronary angiography revealed that one graft was occluded at 2.5 years postoperatively because of flow competition, the grafts in all other patients remained patent during the follow-up period. The graft patency rate at the follow-up period was 80.0% (4/5 grafts).

**Figure 2:**
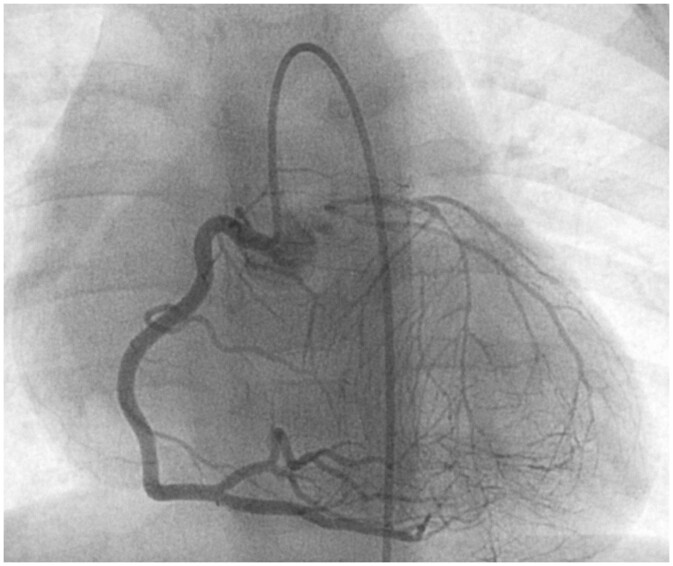
Results of preoperative coronary angiography in Case 4. The left coronary artery could not be catheterized directly because the left main coronary artery was occluded. When we catheterized the right coronary artery, the left coronary artery was lightly contrasted. LCA: left coronary artery.

**Figure 3:**
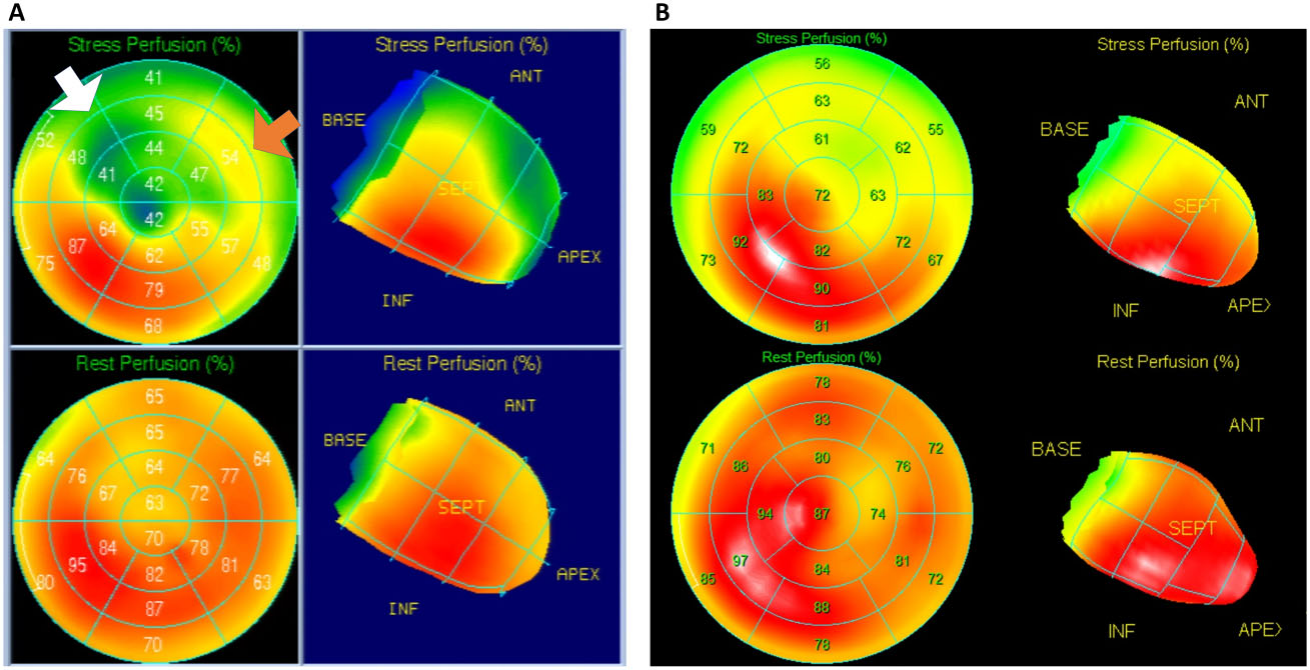
Results of preoperative and postoperative Quantitative Perfusion SPECT in Case 4. (**A**) Preoperative Tc-TF scintigraphy. This examination documents a reversible perfusion defect at rest (bottom rows) and during stress (top rows). The polar plots depict the extent of the ischaemic area in the anterior (white arrow) and lateral left ventricular walls (brown arrow). (**B**) Postoperative TI scintigraphy. Perfusion polar maps during stress (top rows) and at rest (bottom rows) show remarkably improved myocardial perfusion. SPECT: single-photon emission computed tomography; Tc-TF: technetium-tetrofosmin; TI: thallium.

**Figure 4:**
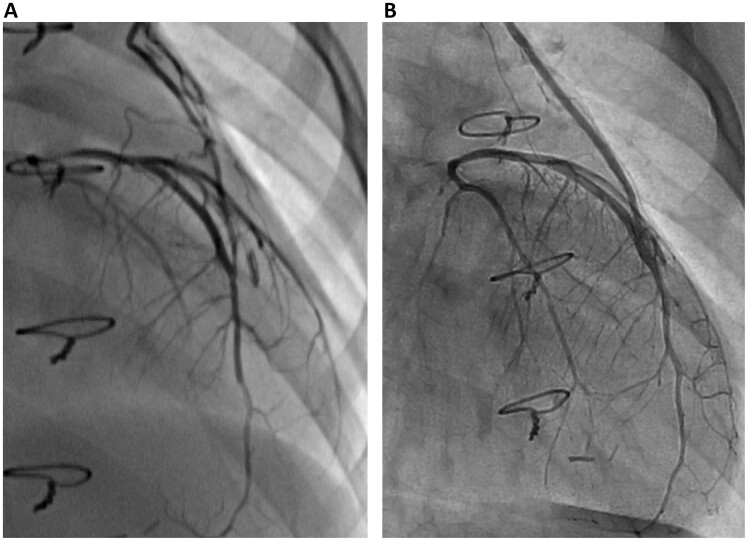
Results of postoperative and follow-up coronary angiography in Case 4. (**A**) Postoperative coronary angiography revealed a patent graft. (**B**) Follow-up coronary angiography revealed graft patency at 17 months postoperatively. These results revealed that the left internal thoracic artery graft remained patent during the follow-up period. LITA: left internal thoracic artery.

**Figure 5:**
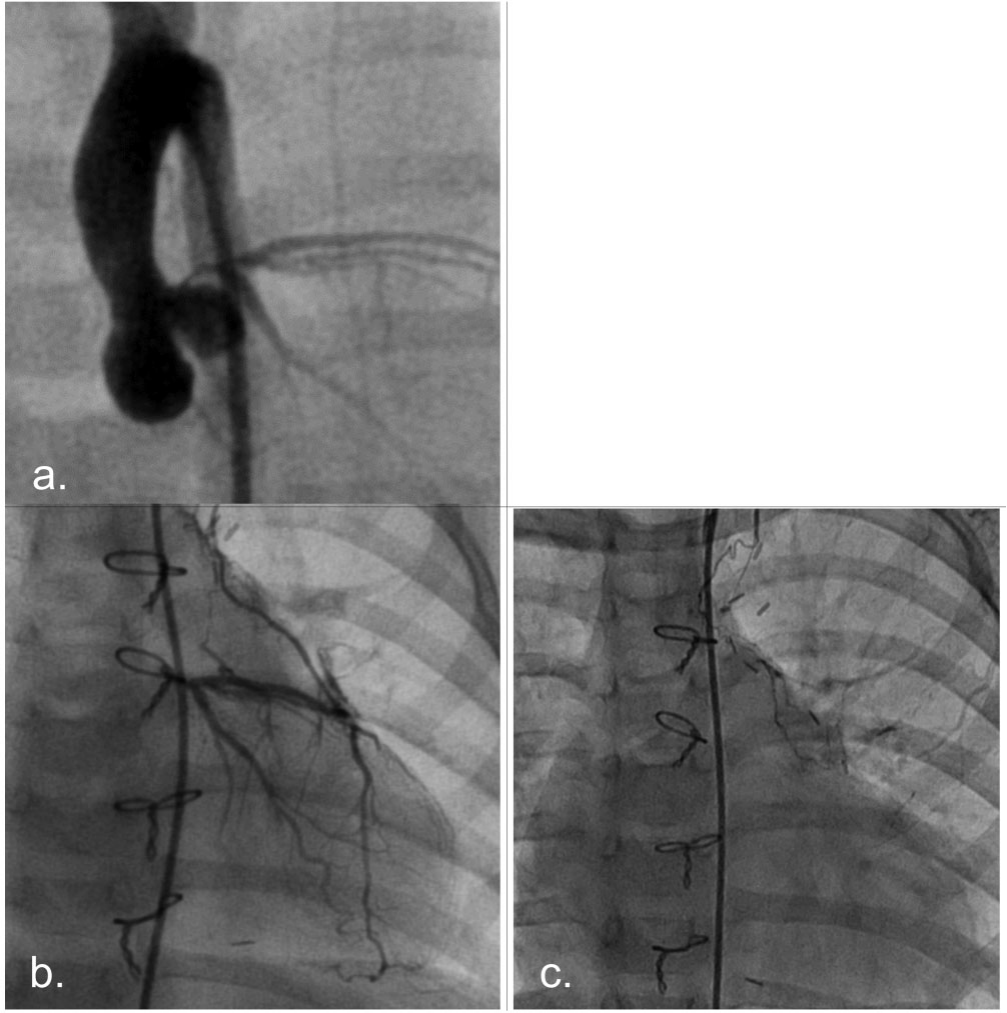
Results of preoperative, postoperative and follow-up coronary angiography in Case 1. (**A**) Preoperative coronary angiography: left main coronary artery stenosis is observed. (**B**) Postoperative coronary angiography: the left internal thoracic artery graft is patent. (**C**) Follow-up angiography performed at 2.5 years postoperatively: the left internal thoracic artery is occluded. LITA: left internal thoracic artery.

**Figure 6:**
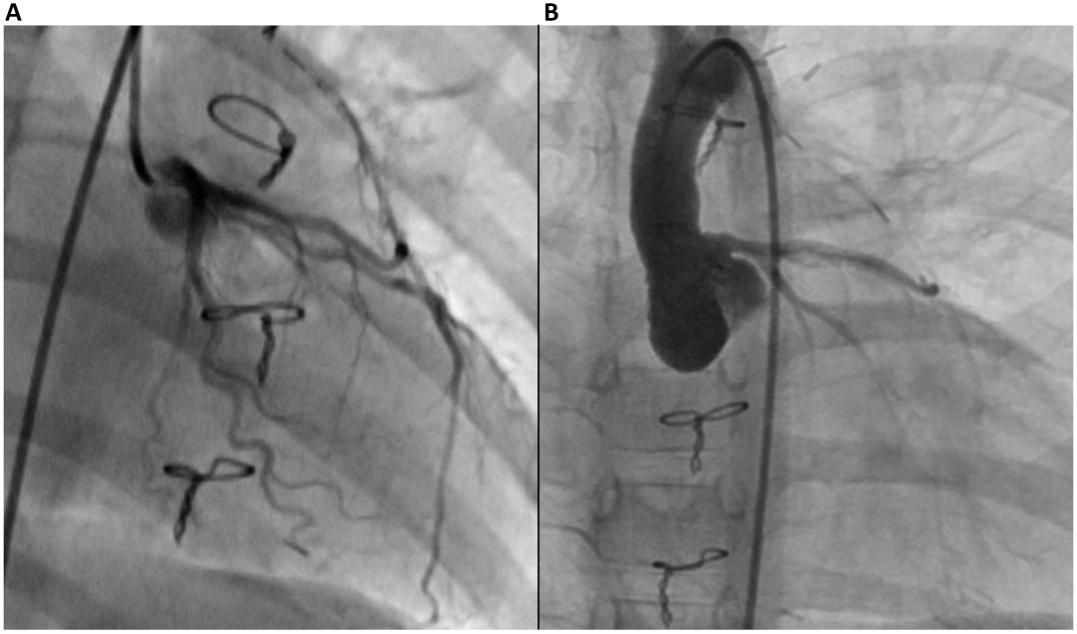
Coronary angiographies were performed at 2.5 and 4.5 years postoperatively in Case 1. (**A**) Follow-up coronary angiography performed at 2.5 years postoperatively. The left coronary artery flow increased. The left internal thoracic artery was contrasted retrogradely from the native coronary artery flow. (**B**) Follow-up coronary angiography performed at 4.5 years postoperatively. Restenosis of the left main coronary artery was not observed. LITA: left internal thoracic artery.

Figures 2–4 depict the results of Case 4, in which the patient was a 3-year-old girl with congenital LMCA atresia and mitral regurgitation. CABG and mitral valve repair were performed in this patient, and no complications or ischaemic symptoms were reported postoperatively. Myocardial scintigraphy showed a postoperative left ventricular (LV) myocardial perfusion improvement in the territory of the left coronary artery compared to that preoperatively (Fig. 3). Figure 4 shows that the LITA graft was patent at 1 and 17 months postoperatively. In contrast, Figs 5 and 6 demonstrate the graft occlusion in Case 1, in which the patient was a 6-month-old girl with LMCA stenosis and supravalvular aortic stenosis (SAS) (Fig. 5A). We performed CABG and aortoplasty (Doty procedure). Postoperative coronary angiography revealed that the graft was patent (Fig. 5B); however, at 2.5 years later, the LITA was barely contrasted antegradely (Fig. 5C) because of increased blood flow in the native left coronary artery (Fig. 6A). During the follow-up period, LMCA restenosis did not occur (Fig. 6B), and the patient’s LVEF was stable at >80%. Therefore, we did not perform any reinterventions for the coronary artery anomaly during the follow-up period.

## DISCUSSION

There are several types of congenital coronary artery anomalies; however, the overall incidence is very low (0.2–1.3% of patients who undergo coronary angiography) [8]. Among such anomalies, some diseases, including LMCA atresia or stenosis can cause sudden death in infants and children [9, 10]. In addition to CABG, various other surgical approaches have been previously reported for these coronary artery anomalies, such as patch plasty with an autologous pericardium, an azygos vein patch or a saphenous vein for LMCA stenosis or atresia [11, 12]. However, because of the lack of available data regarding clinical experiences and postoperative outcomes, it remains unclear which surgical treatment is optimal for the treatment of congenital coronary artery anomalies. We believe that CABG can be used appropriately for coronary revascularization in various types of coronary artery anomalies. This is because CABG is one of the most popular procedures for coronary revascularization in adult coronary artery disease [13] and internal thoracic artery (ITA) bypass grafting has been proven to yield excellent long-term clinical outcomes [14, 15]. Although target vessels in infants and young children are much smaller than those in adults, some studies have recommended CABG for coronary revascularization in infants and children with coronary artery disease [1–3]. Therefore, we believe that younger age is not always a risk factor for reduced graft patency rates. Furthermore, ITA patency is far superior to saphenous vein graft patency for any coronary arteries because the ITA has the potential to grow longitudinally and circumferentially with the child’s somatic growth after coronary artery bypass surgery [16, 17]. Therefore, ITA–left anterior descending artery bypass grafting can be a suitable strategy for coronary revascularization in infants and young children.

After CABG, postoperative LVEF is an important determinant of the outcome in children [2]. Previous studies have shown that poor LV function tends to be associated with sudden death after paediatric CABG [2, 18]. In the present study, the postoperative LVEF was >60% in all cases. Thus, sufficient LV function may be attributed to the occurrence of no deaths or cardiac events. In contrast, postoperative LVEF was not improved significantly compared with preoperative LVEF. Particularly, Cases 1–3, which were accompanied by congenital aortic stenosis and SAS, did not show any improvement in LVEF postoperatively. This is because hyperdynamic LV function occurs with high LVEF preoperatively due to increased LV pressure overload [19]. Meanwhile, LVEF returned to normal postoperatively because of the release of aortic stenosis or SAS, as LVEF is affected not only by contractility but also by preload and afterload [20, 21]. Therefore, despite the lack of significant differences in LVEF improvement postoperatively, the good LV function (LVEF >60%) led to a good postoperative outcome in the follow-up period.

Several studies have reported favourable short- and mid-term outcomes for surgical approaches that implemented LMCA angioplasty with aortoplasty to correct SAS with LMCA involvement [22, 23]. However, no studies have reported the long-term outcomes of such LMCA angioplasty. In adult patients with LMCA stenosis, the postoperative outcome of surgical angioplasty is inferior to that of CABG. Furthermore, involvement of the LMCA bifurcation leads to poorer outcomes in surgical angioplasty [24, 25]. Therefore, as the distal bifurcation is much closer to the LMCA ostium in infants and young children than in adults, the long-term outcomes of LMCA angioplasty to treat LMCA lesions with SAS are questionable. In contrast, regarding CABG, even when ITA grafts have been judged as occluded or non-functioning postoperatively (as in Case 1), a relatively high incidence of recanalization can be expected in paediatric CABG compared with that in adult CABG [26]. Thus, although there was a risk of flow competition postoperatively, we considered CABG as the optional strategy for LMCA lesion with SAS rather than LMCA angioplasty.

This study revealed that CABG is a valuable strategy for coronary revascularization for congenital coronary artery anomalies in infants and young children. Some studies have described cases of CABG for congenital coronary artery anomalies [5–7]. However, to the best of our knowledge, this is the first report of the short- and mid-term postoperative outcomes of CABG for coronary artery anomalies in infants and young children. Furthermore, even if the short- and mid-term patency and postoperative outcomes are satisfactory, a careful follow-up examination is necessary because the long-term patency and outcomes remain unknown.

### Limitations

This study has some limitations. First, it had a retrospective design with a small number of enrolled patients. Second, the follow-up duration of the cases was not long enough to allow for a definitive conclusion regarding the effectiveness of CABG in infants and young children with congenital coronary artery anomalies. Thus, it is necessary to consider longer follow-up periods in future studies involving such evaluations. However, the present study is the first to evaluate the outcome of CABG for coronary artery anomalies in infants and young children with at least mid-term follow-up. Moreover, Kitamura *et al.* [2] reported that ITA grafts that were patent at the early-term follow-up after surgery remained so for a long-term follow period in children. Therefore, although this study only had a mid-term follow-up period, it provides valuable insights into congenital coronary artery anomalies. Finally, all procedures were performed at a single centre by a single surgeon, thereby introducing the possibility of selection bias. Hence, future studies must involve a larger number of patients and multiple centres across different regions.

## CONCLUSION

We revealed that CABG is a useful method for coronary revascularization in infants and young children with congenital coronary artery anomalies. The short- and mid-term patency rates and surgical outcomes were satisfactory. However, continuous follow-up examination is warranted because the long-term patency and outcomes remain unknown.

**Conflict of interest:** none declared.

## Data availability statement

The data underlying this article will be shared on reasonable request to the corresponding author.

## Author contributions

**Yu Hohri:** Writing—original draft. **Masaaki Yamagishi:** Conceptualization. **Yoshinobu Maeda:** Supervision. **Satoshi Asada:** Resources. **Hisayuki Hongu:** Resources. **Satoshi Numata:** Supervision. **Hitoshi Yaku:** Project administration.

## Reviewer information

Interactive CardioVascular and Thoracic Surgery thanks Katarzyna Januszewska, Hitendu Hasmukhlal Dave and the other anonymous reviewers for their contribution to the peer review process of this article.

## Notes

Presented at the 35th Annual Meeting of the European Association for Cardio-Thoracic Surgery, Barcelona, Spain, 13–16 October 2021.

## ABBREVIATIONS

CABG: Coronary artery bypass grafting
ITA: Internal thoracic artery
LITA: Left internal thoracic artery
LMCA: Left main coronary artery
LV: Left ventricular
LVEF: Left ventricular ejection fraction
SAS: Supravalvular aortic stenosis
